# Supplementary material for: Universally bistable shells with nonzero Gaussian curvature for two-way transition waves
Source: Nat Commun. 2021 Jan 29;12:695. doi: 10.1038/s41467-020-20698-9 (PMC7846611; doi:10.1038/s41467-020-20698-9)
Supplement: Supplementary file 1 — Supplementary Information [file 41467_2020_20698_MOESM1_ESM.pdf]

Supplementary Information

Nikolaos Vasios[1], Bolei Deng[1], Benjamin Gorissen[1] and Katia Bertoldi[1,*]

[1]J. A. Paulson School of Engineering and Applied Sciences,
Harvard University, Cambridge, MA 02138, USA
*To whom correspondence should be addressed;
E-mail: bertoldi@seas.harvard.edu

Supplementary Note 1: Our Thick Shells

In this section we provide details on the geometry, fabrication, testing and Finite Element modeling of the thick shells considered in this study.

1.1 Geometry

1.1.1 Single Doubly Curved Shells

The geometry of the doubly curved shells considered in our study is based on the 360° revolution of the height profile, h, given by

$$h = \begin{cases} H\left[1 + 2\left(\frac{r}{R}\right)^3 - 3\left(\frac{r}{R}\right)^2\right], & r \in [0, R] \\ 0, & r \in [R, R+S] \end{cases}, \tag{1}$$

where H is the maximum shell height at $r = 0$, R is the shell radius and S denotes the length of a flat portion added at the base of the cap to facilitate the enforcement of boundary conditions in experiments and simulations (see black dashed line in Fig. 1). The final geometry of the single doubly curved shells is obtained by offsetting $h(r, R, H, S)$ at a distance equal to the shell thickness T (see Fig. 1). Note that the particular expression for the profile of the shell $h(r, R, H, S)$ can be obtained starting from a 3rd order polynomial by demanding that the maximum height of the shell at $r = 0$ is H and the height of the shell at the base of the profile ($r = R$) drops to zero,

$$h(0, R, H, S) = H, \qquad h(R, H, S) = 0. \tag{2}$$

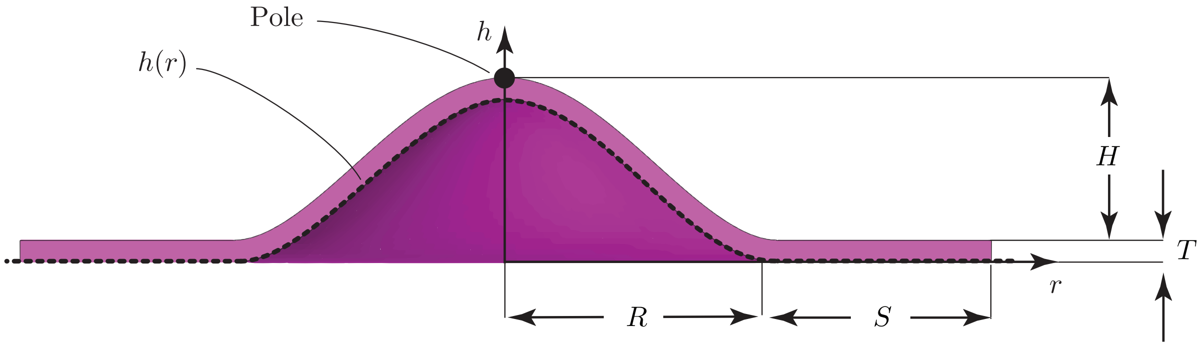

Supplementary Figure 1: Schematic of the single doubly curved shell considered in this study. The dashed line indicates the profile h. The shell is obtained by offsetting the the profile at a distance equal to the nominal thickness T.

Finally, we demand that the gradient of the profile at the top ($r = 0$) and base ($r = R$) is also equal to zero,

$$\left.\frac{dh(r, R, H, S)}{dr}\right|_{r=0} = 0 \quad , \quad \left.\frac{dh(r, R, H, S)}{dr}\right|_{r=R} = 0 \tag{3}$$

The former constraint is a result of the axial symmetry of the profile whereas the latter ensures that there is a continuous transition from the shell profile to the flat portion.

1.1.2 Universally Bistable Doubly Curved Shells

The universally bistable doubly curved shells considered in this study are obtained by combining two identical single shells, the geometry of which is described in Section 1.1.1. We first place the two identical single shells with radius R, height H and thickness T (see Fig. 2a) as mirrored images of each other (see Fig. 2b) and we then compress the shells by two rigid plates until they elastically deform into a flat configuration (see Fig. 2c). Next, we glue the two shells in the deformed flat state, thus forming a single doubly curved shell for which the the stress-free configuration has been eliminated. Note that the newly formed doubly curved shell will naturally rest in a new stable state, and will in general have a different height profile from the one of the single shells that comprise it (see Fig. 2d).

All universally bistable shells fabricated and tested in this study are comprised of two identical single shells for which the geometric parameter values are presented in Table 1.

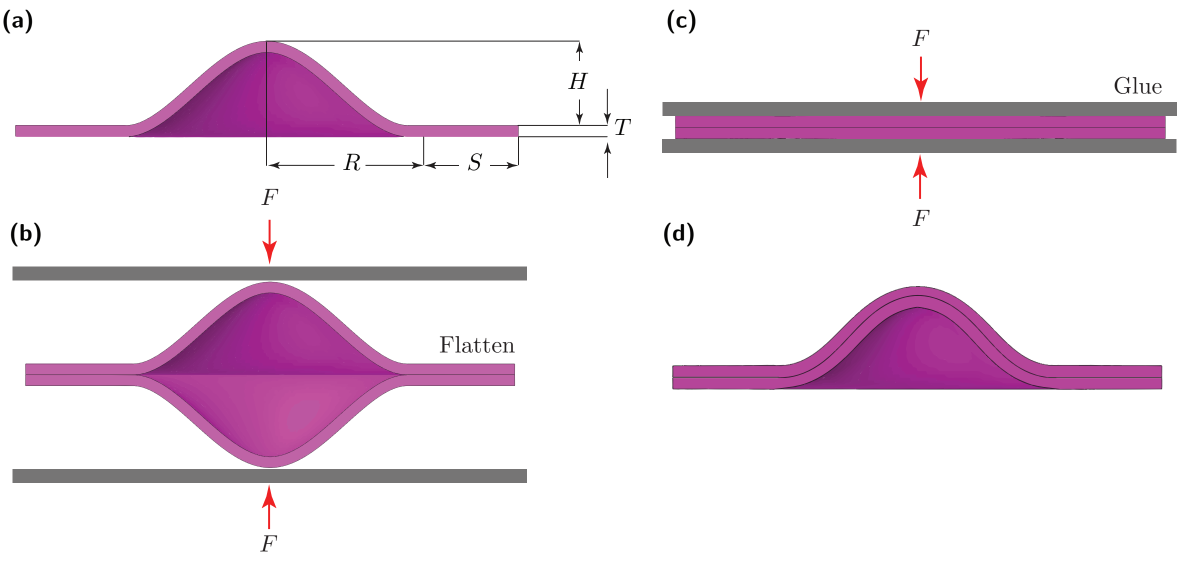

Supplementary Figure 2: Geometry and construction of universally bistable shells. (a) The universally bistable shells comprise two doubly curved single shells. (b) The two single shells are placed as mirrored images of each other. (c) The two shells are elastically deformed into a flat configuration and glued together. (d) The newly formed doubly curved shell rests in a new stable state.

1.2 Fabrication

All shells tested in this study are made of Elite Double 8 silicone rubber (Zhermack) and were casted using the two-part mold shown in Fig. 3. The mold was 3d printed in Vero-clear using an Objet Connex 500 printer (Stratasys).

1.2.1 Single Doubly Curved Shells

Our single shells can be fabricated using the following 3 steps (see Fig. 4):

Step 1: expose all inner surfaces of the mold to Ease Release 200 spray (Mann Release Technologies) to facilitate the process of removing the cured silicone rubber.
Step 2: pour a mixture of equal amounts of Elite Double 8 base and catalyst (here made using the Zhermack Doublemix mixer) into Part A of the mold.
Step 3: slowly place part B of the mold on top of part A, allowing for any excess silicone rubber to flow out of the mold and cure for 20 minutes.
All shells fabricated and tested in this study have Radius $R = 25.4$ mm, height $H = 15$ mm, thickness $T = 2mm$ and a flat portion of length $S = 20$ mm (see Table 1)

1.2.2 Universally Bistable Shells

Our thick shells can be fabricated using the following 9 steps (see Fig. 5):

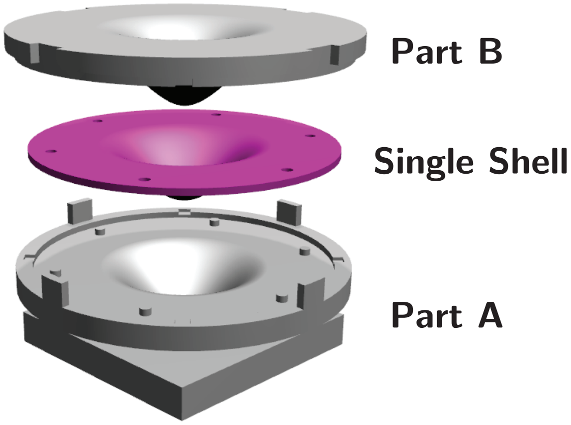

Supplementary Figure 3: The 3D printed mold used for the fabrication of all single shells.

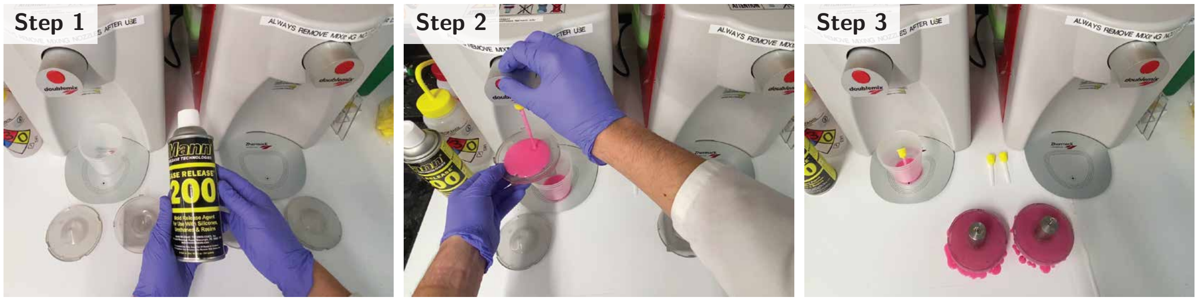

Supplementary Figure 4: The 3 steps for the fabrication of the single doubly curved shells.

Radius (R)	25.4 mm
Height (H)	15 mm
Thickness (T)	2 mm
Flat Length (S)	20 mm

Table 1: Values of the geometric parameters for the shells fabricated and tested in this study.

Step 1: expose all inner surfaces of the molds to Ease Release 200 spray (Mann Release Technologies) to facilitate the process of removing the cured silicone rubber.
Step 2: pour a mixture of equal amounts of Elite Double 8 base and catalyst (here made using the Zhermack Doublemix mixer) into Part A of the mold.
Step 3: slowly place part B of the mold on top of part A, allowing for any excess silicone rubber to flow out of the mold and cure for 20 minutes.
Step 4: remove the single shell from its molds peeling off any excess silicone. Repeat the process to fabricate two shells.

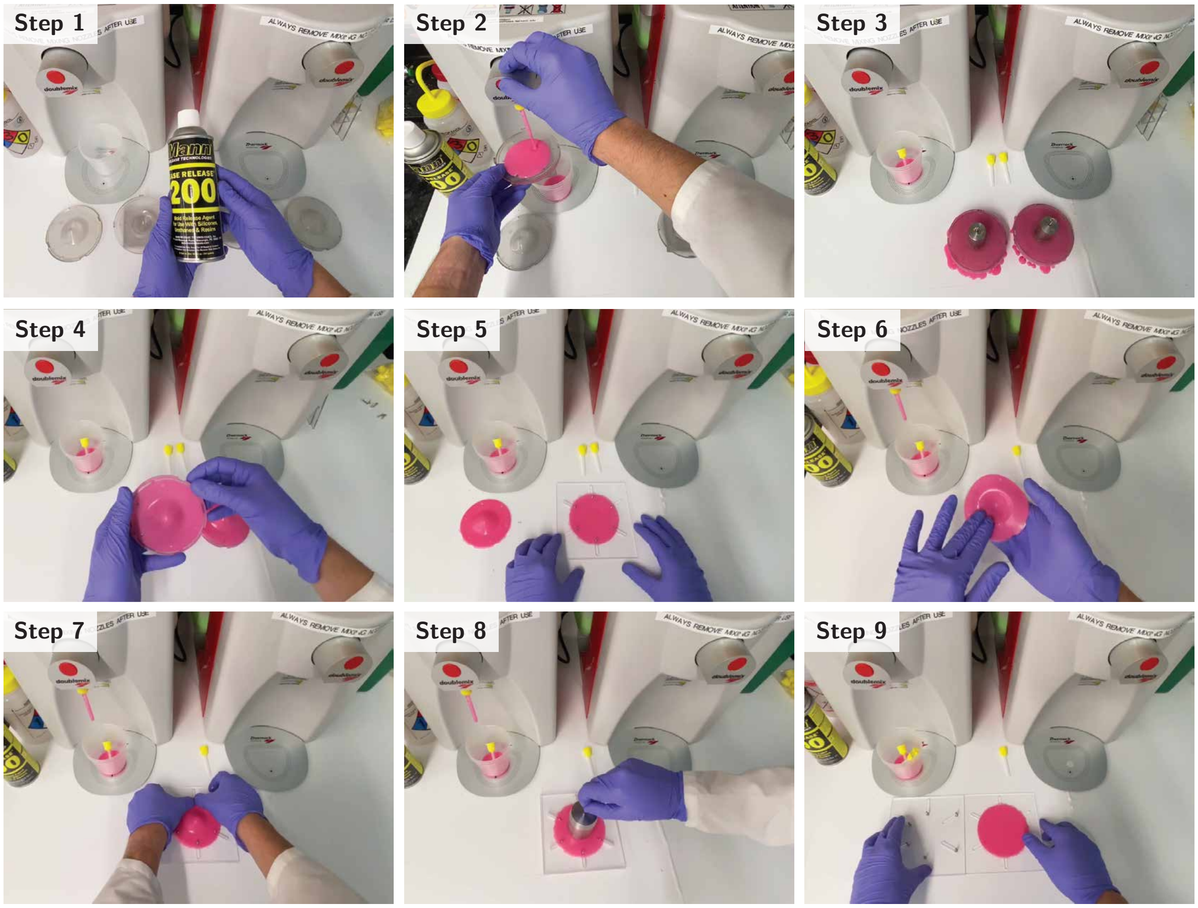

Supplementary Figure 5: The 9 steps for the fabrication of the universally bistable shells.

Step 5: place one of the shells upside down on a flat rigid surface.
Step 6: gently spread a thin layer of Elite Double 8 on the inner surface of the second shell to glue the two shells together.
Step 7: place and align the second shell directly on top of the upside-down shell.
Step 8: flatten the two shells with a rigid plate. Place a small weight on top of the rigid surface to ensure that the two shells remain in contact while the silicone rubber cures.
Step 9: cure for 20 minutes.

1.3 Testing

In order to characterize the quasi-static response of our shells we conducted experiments to determine their pressure-volume relationship and quantify the displacement of their pole upon inflation and deflation. We tested all shells using the the custom-made setup shown in Fig. 6a, which is made using a clear cast acrylic tube (McMaster Carr part number: 8532K46) and laser-cut acrylic plates (McMaster Carr part number: 1178T12). In all our tests, to eliminate the effect of fluid compressibility, we load the shells by supplying water at a constant rate of 30 mL/min with a syringe pump (Pump 33DS, Harvard Apparatus).

Pressure-Volume. The pressure-volume curve of our shells is obtained by monitoring the pressure during the tests with a pressure sensor (MPXV7025DP by NXP USA). Note that to eliminate the influence of gravity, for these tests we submerge the entire custom-made setup shown in Fig. 6a in a water tank (see Fig. 6b).

Displacement-Volume. In order to monitor the displacement of the shell's pole during inflation and deflation, we use a green-colored pin attached to the shell (see Fig. 7c-g and Fig. 8c-g). We inflated and deflated the shells and recorded videos of their deformation which we processed to extract the displacement history of the shells' center point. Specifically, for each recorded frame, a custom Matlab script was used to monitor and log the location of the green colored pin, from which we were able to infer the displacement of the shells' center point. Note that to eliminate the effect of viscous forces to the motion of the green-colored pin we performed these tests at ambient pressure without having the shells submerged in the water tank.

1.3.1 Results

In Figs. 7a and 8a we report the evolution of the pressure p recorded when prescribing a change in volume ΔV for a single doubly curved shell (with geometric parameters described in Table 1) and a universally bistable shell (comprising two shells with geometric parameters described in Table 1), respectively. For both structures the inflation (blue lines) and deflation (red lines) pressure-volume curves do not coincide owing to the hysteretic behavior of the shell's deformation. All curves are extremely non-linear featuring a local-maximum pressure value during inflation $p_{i,max}$, and a local minimum pressure value during deflation $p_{d,min}$. The fact that the pressure-volume curves cross the horizontal axis ($p = 0$) more than once indicates that the shells have two stable states, their initial state and the inverted state. The positive pressure limit point during inflation ($p_{i,max}$) corresponds to the pressure required to invert the shell to its second stable state. Moreover, the pressure limit point during the deflation of the shell ($p_{d,min}$) corresponds to the negative pressure required to push the shell back to its initial state. Note that for the universally

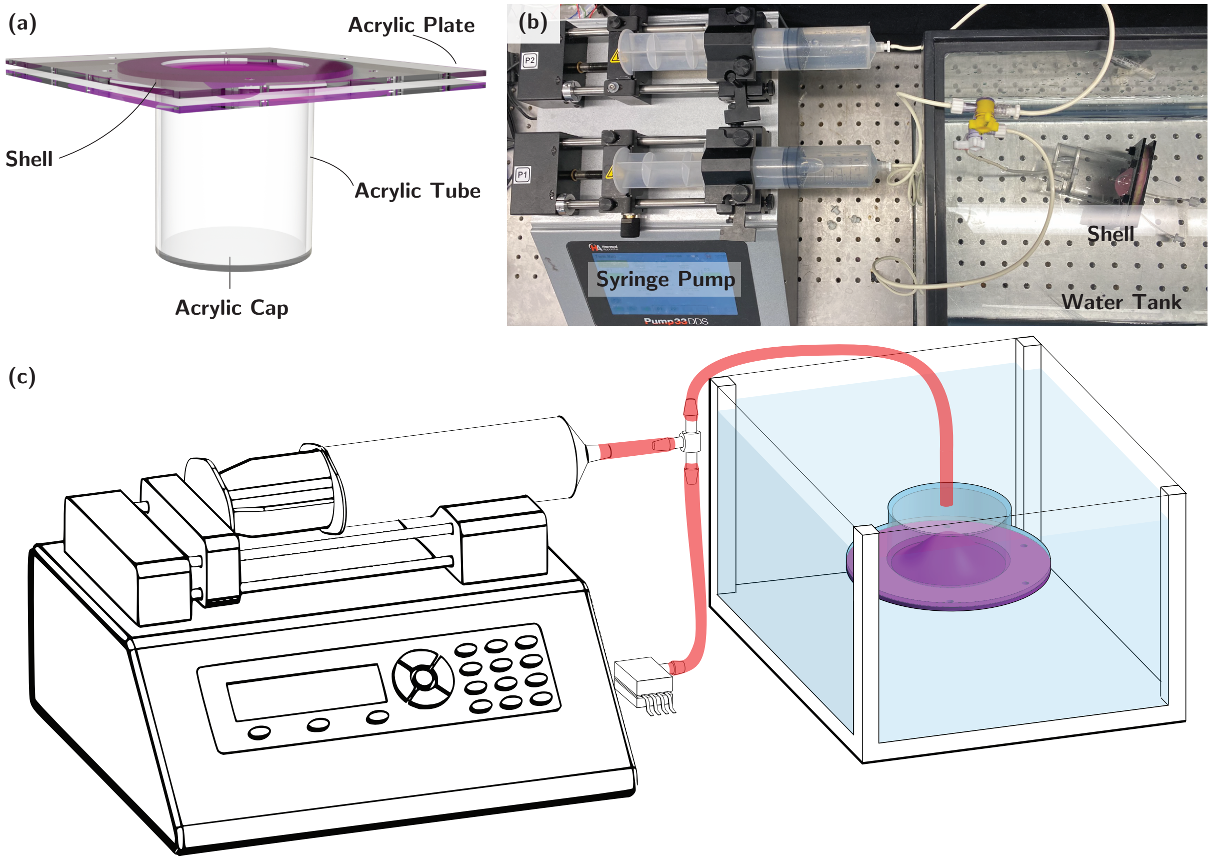

Supplementary Figure 6: Experimental setup for the quasi-static testing of shells subjected to inflation and deflation. (a) The custom apparatus comprised of clear cast acrylic tube and laser-cut acrylic plates. (b) The syringe pump used to inflate/deflate she shells while fully submerged in the water tank. (c) Schematic of the experimental setup used to quasi-statically inflate and deflate the universally bistable shells using water, while being submerged in a water tank.

bistable shells $|p_{i,max}| \sim |p_{d,min}|$, so that the pressure required to transition between the stable states is identical owing to the "stress-symmetry" between them. Differently, for the single shell $|p_{i,max}| > |p_{d,min}|$ suggesting that the input energy required to invert the shell is larger than the corresponding energy required to push the shell back to its initial state.

In Figs. 7b and 8b we report the evolution of the vertical component of the pole displacement, u_{pole}, extracted when prescribing a change in volume ΔV. Also in this case we find that the displacement-volume curves during inflation (shown in blue in Fig. 7b and Fig. 8b) do not coincide with the deflation curves (shown in red in Fig. 7b and Fig. 8b). Furthermore, our results indicate that at the volume change ΔV that corresponds to

the two pressure limit points $p_{i,max}$ and $p_{d,max}$, the poles undergo a sudden and large displacement, a feature typical of snap-through instabilities.

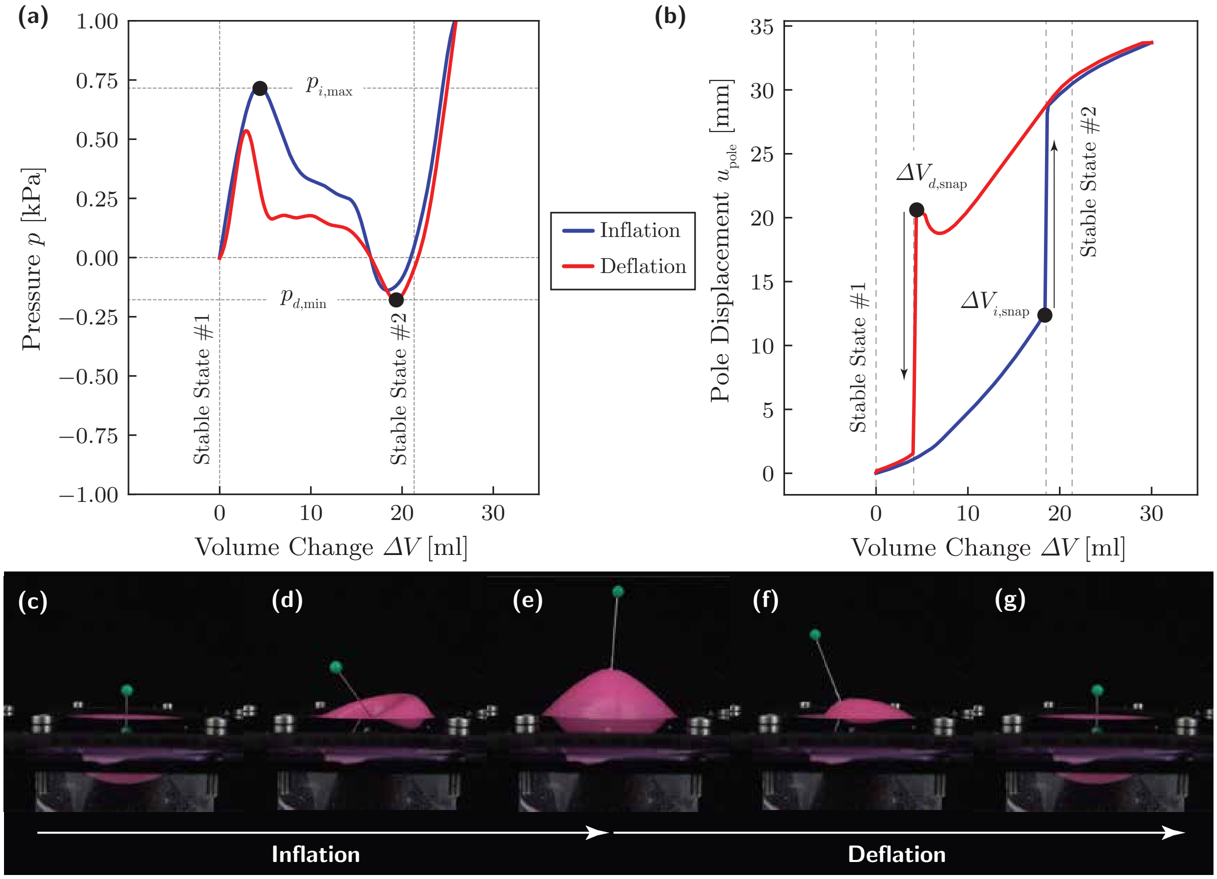

Supplementary Figure 7: Testing of doubly curved shells. (a)-(b) The experimental pressure-volume and displacement-volume relationships. (c)-(e) Snapshots of the shell's deformation during inflation and deflation highlighting the pole displacement.

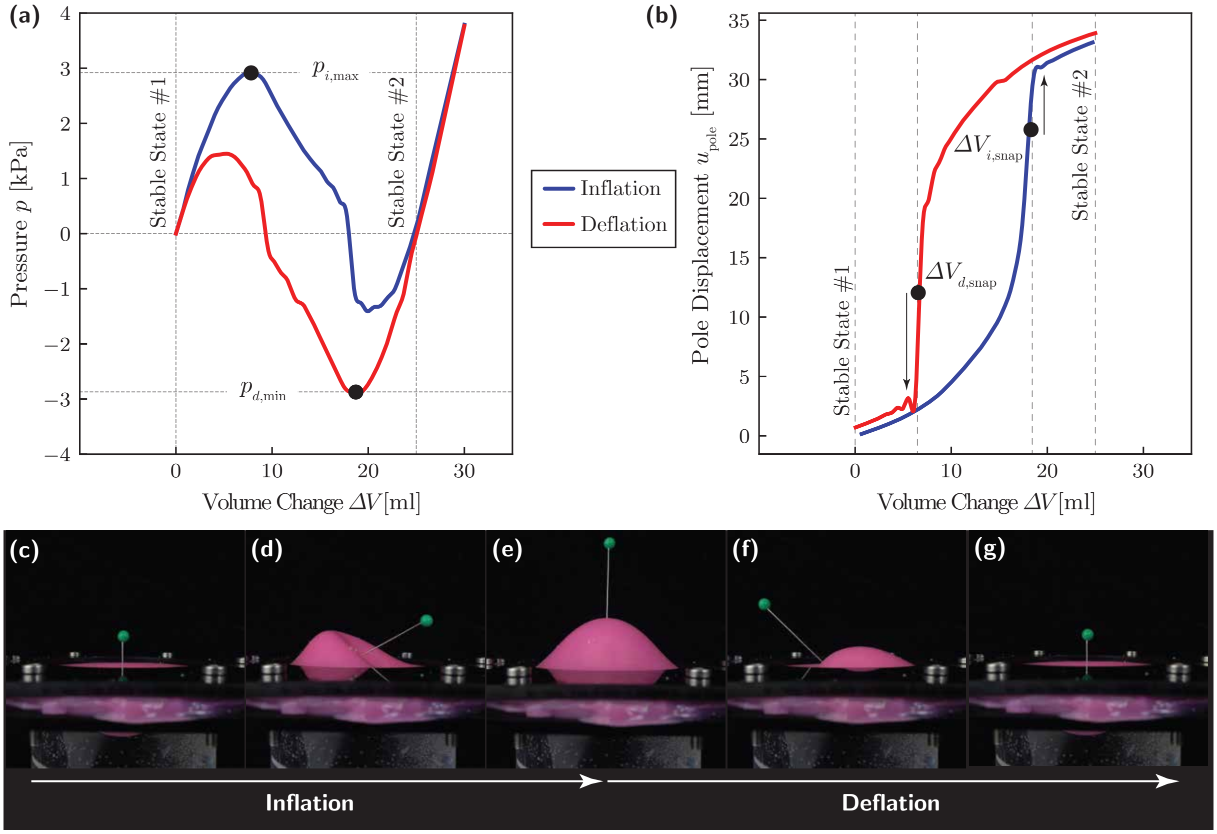

Supplementary Figure 8: Testing of universally bistable shells. (a)-(b) The experimental pressure-volume and displacement-volume relationships. (c)-(e) Snapshots of the shell's deformation during inflation and deflation highlighting the pole displacement.

1.4 Finite Element Modeling

In an effort to better understand the effect of geometric parameters on the response of our shells, we performed Finite Element simulations using the commercial non-linear Finite Element software Abaqus (Dassault Systemes, SIMULIA). In all our analyses we modeled Elite Double 8 as an incompressible Neo-Hookean solid with an initial shear modulus $\mu = 83$kPa [?]. We constructed half shell models and meshed them using 8-noded fully integrated hybrid linear brick elements (C3D8H) as shown in Fig. 9a. To capture the response of the full shell geometry with our half-shell models, we imposed symmetry boundary conditions $u_z = 0$ on the flat face of the models (see Fig. 9b). The models were inflated and deflated by controlling volume through the fluid filled cavity interaction and rigid body motions were eliminated by fixing all displacement components for the outer-rim of all shells (see Fig. 9c).

1.4.1 Single Doubly Curved Shells

The finite element analyses for the inflation and deflation of the single doubly curved shells consist of the following steps:

Step 1: Perform a static step to pressurize the shells by imposing a pressure on their inner surface equal to $p = 10^{-7} \times \mu$.
Step 2: Perform a Frequency Analysis about the pressurized state to obtain the first eigenmode for the shell (see Fig. 9d).
Step 3: Perturb the initial shell geometry through

$$\boldsymbol{x}_p^{(i)} = \boldsymbol{x}^{(i)} + \lambda \boldsymbol{u}_1^{(i)},$$

where $\boldsymbol{x}_p^{(i)}$ is the perturbed coordinate vector for node i, $\boldsymbol{x}^{(i)}$ is the initial coordinate vector for node i, $\boldsymbol{u}_1^{(i)}$ is the displacement vector corresponding to the first frequency mode for node i and $\lambda = 5 \times 10^{-5}$ is a scalar amplification factor to ensure that the perturbation amount does not significantly alter the base geometry (see Fig. 9e).
Step 4: Perform a Dynamic Implicit simulation with a duration of $t = 60$s where the shell is being inflated for $t \in [0, 30]$s and deflated for $t \in [30, 60]$s. Despite the quasi-static nature of the experiments, we used dynamic implicit simulations to better capture the snap-through instabilities that are triggered during the inflation and deflation of the shells. Note that, to eliminate spurious vibrations during the inflation/deflation of the shells, in these analyses we use a stiffness proportional Rayleigh damping parameter $\beta_R = 0.01$.

1.4.2 Universally Bistable Shells

The finite element analyses for the inflation and deflation of the universally bistable shells consist of the following steps:

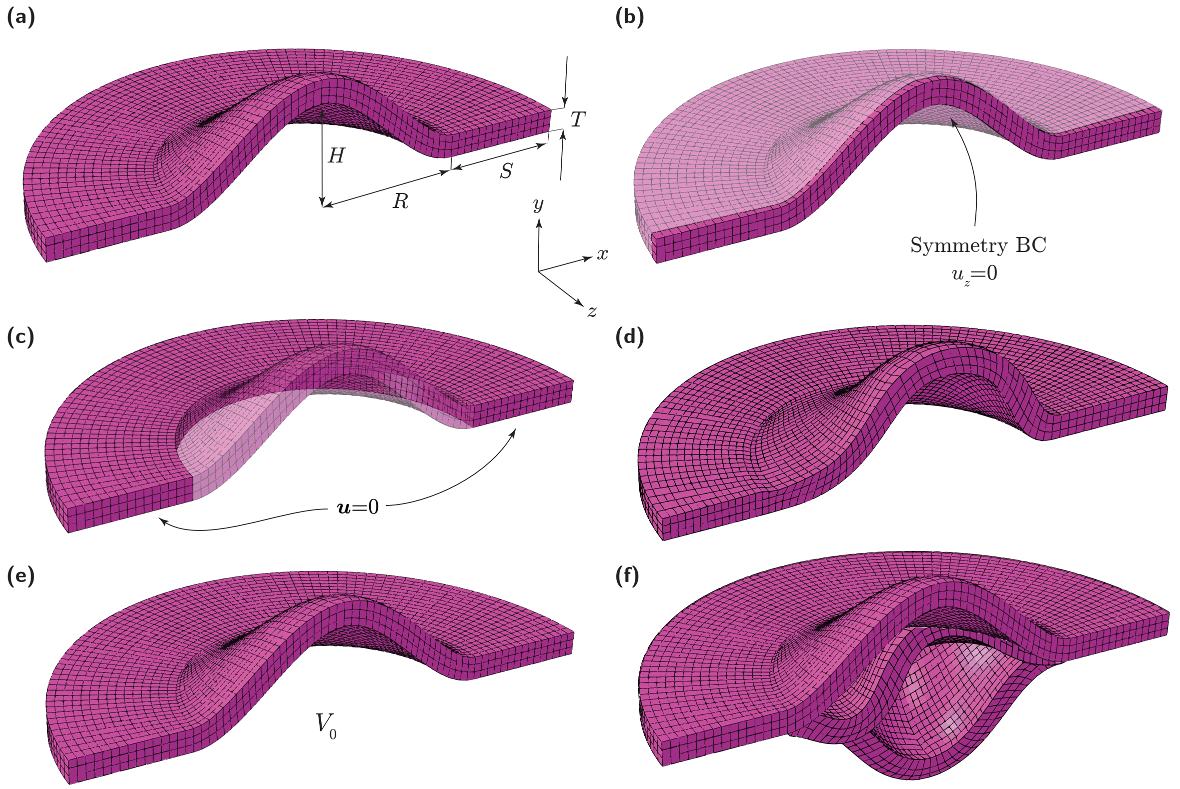

Supplementary Figure 9: Finite element modeling of single doubly curved shells. (a) The symmetric base half-shell geometry. (b) The region on which the symmetry boundary condition is applied. (c) The region on which the zero displacement boundary condition is applied. (d) The first frequency mode (amplified) as computed from the frequency analysis step. (e) The perturbed shell geometry. (f) Snapshots of the shell's deformation during inflation and deflation.

Step 1: Starting with two identical single shells positioned as mirrored images of one another (see Fig. 10a), perform a quasi-static step in which both shells are elastically deformed until their inner surfaces become perfectly flat. Specifically, to each node on the inner surface of the shell we prescribe a displacement in vertical direction $u = -h(r)$.
Step 2: Define a new model with initial geometry identical to the deformed geometry of the two single shells in their flat configuration. Import the stress field due to the elastic flattening of the single shells and impose it as an initial condition for the new model.
Step 3: Enforce a tie constraint for all nodes located in the inner surface of the two single shells to form the universally bistable shell.
Step 4: Perform a quasi-static step to perturb the geometry of the universally bistable shell from the flat configuration by supplying a small pressure load ($p = 10^{-2} \times \mu$, see Fig. 10b) and subsequently remove the pressure load, allowing the universally bistable shell to rest in its stable state (see Fig.10c).

Step 5: Perform a Frequency Analysis step to compute the first eigenmode for the universally bistable shell (see Fig.10d).
Step 6: Perturb the universally bistable shell geometry through

$$\boldsymbol{x}_p^{(i)} = \boldsymbol{x}^{(i)} + \lambda \boldsymbol{u}_1^{(i)},$$

where $\boldsymbol{x}_p^{(i)}$ is the perturbed coordinate vector for node i, $\boldsymbol{x}^{(i)}$ is the initial coordinate vector for node i, $\boldsymbol{u}_1^{(i)}$ is the displacement vector corresponding to the first frequency mode for node i and $\lambda = 5 \times 10^{-5}$ is a scalar amplification factor to ensure that the perturbation amount does not significantly alter the base geometry.
Step 7: Perform a Dynamic Implicit simulation with a duration of $t = 60$s where the shell is being inflated for $t \in [0, 30]$s and deflated for $t \in [30, 60]$s (see Fig.10e-f). To eliminate spurious vibrations during the inflation/deflation of the shells, use a stiffness proportional Rayleigh damping parameter $\beta_R = 0.01$.

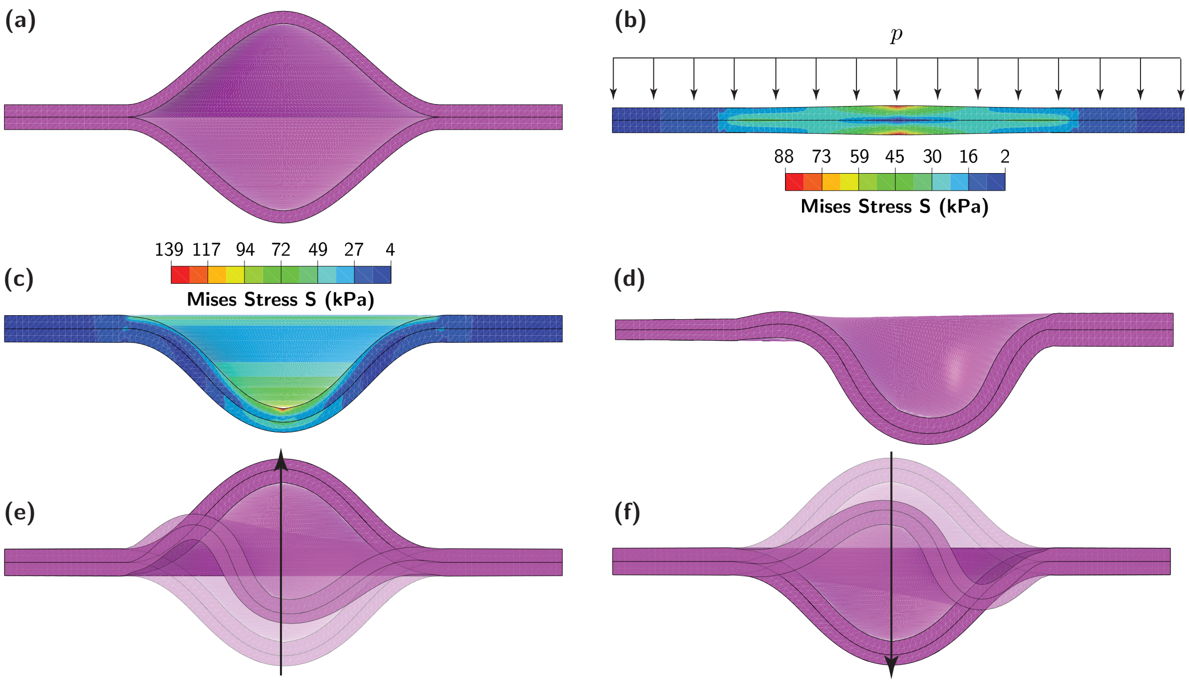

Supplementary Figure 10: Finite element modeling of universally bistable shells. (a) Two single shells are positioned as mirror images of one another. (b) The two single shells elastically deformed until flat, and they are glued in their deformed configuration. (c) The new stable state of the universally bistable shell is not stress-free. (d) The first frequency mode of the universally bistable shell (amplified) as computed from the frequency analysis step. (e)-(f) Inflation-Deflation of the universally bistable shell.

1.4.3 Results

To verify the validity of our Finite Element simulations, we first compared their predictions to the experimental results obtained when inflating and deflating our shells (see experimental results reported in Fig. 7 and Fig. 8). The results reported in Figs. 11 indicate that the FEA simulations can accurately capture the response of both our single doubly curved and universally bistable shells during inflation and deflation. Finally, in Figs. 11c and f we show the evolution of the elastic strain energy U as a function of their pole displacement, u_{pole}, for both inflation and deflation of the shells. We find that the inverted state of the single doubly curved shell is characterized by an energy much higher than that of the initial state. In contrast, for the universally bistable shell U exhibits two energy minima of identical height, suggesting that the shell has two equally preferable stable states. Note that the energy minima for the universally bistable shell correspond have both non-zero elastic strain energy, owing to their fabrication process which eliminates the existence of a stress free state.

Having established the validity of our FEA simulations, we then use them to investigate the effect of the shells' height H (with $H \in [10, 20]$ mm) and thickness T (with $T \in [2, 6]$ mm) on their mechanical response. For each considered geometry, we simulated the inflation and deflation following the steps outlined in Section 1.4.1 and Section 1.4.2 for the single and universally bistable shells, respectively, and extracted their elastic strain energy U as a function of their pole displacement u_{upole} (see Fig. 12b). Next, we identified the minima of U and defined the energy barrier U_b as the energy required to deform the shells from their initial stable state to their inverted state, and the energy release U_r as the energy difference between the inverted state of the shell and its initial stable state.

In Figs. 12c and e and Figs. 12d and f we report contour plots of the normalized energy barrier $U_b/\mu RHT$ and energy release $U_r/\mu RHT$ (μ being the shear modulus of Elite Double 8 and R denoting the shell radius) for the single and universally bistable shells as a function of their height H and thickness T. Three key features emerge from the plots. First, all considered universally bistable shells are bistable (hence their name "universally bistable"), whereas the single shells are bistable only for $T <$4.05mm (the gray region in the contour plots corresponds to geometries for which the shells were found to be monostable and therefore the energy barrier U_b and energy release U_r could not be defined). Second, for both single and universally bistable bistable shells the energy barrier U_b increases as the shell height H and the thickness T become larger. Third, while the energy release E_r for the single bistable shells increases as a function of both H and T, it is zero for all universally bistable shells - an indication that the two energy minima for these system are always identical.

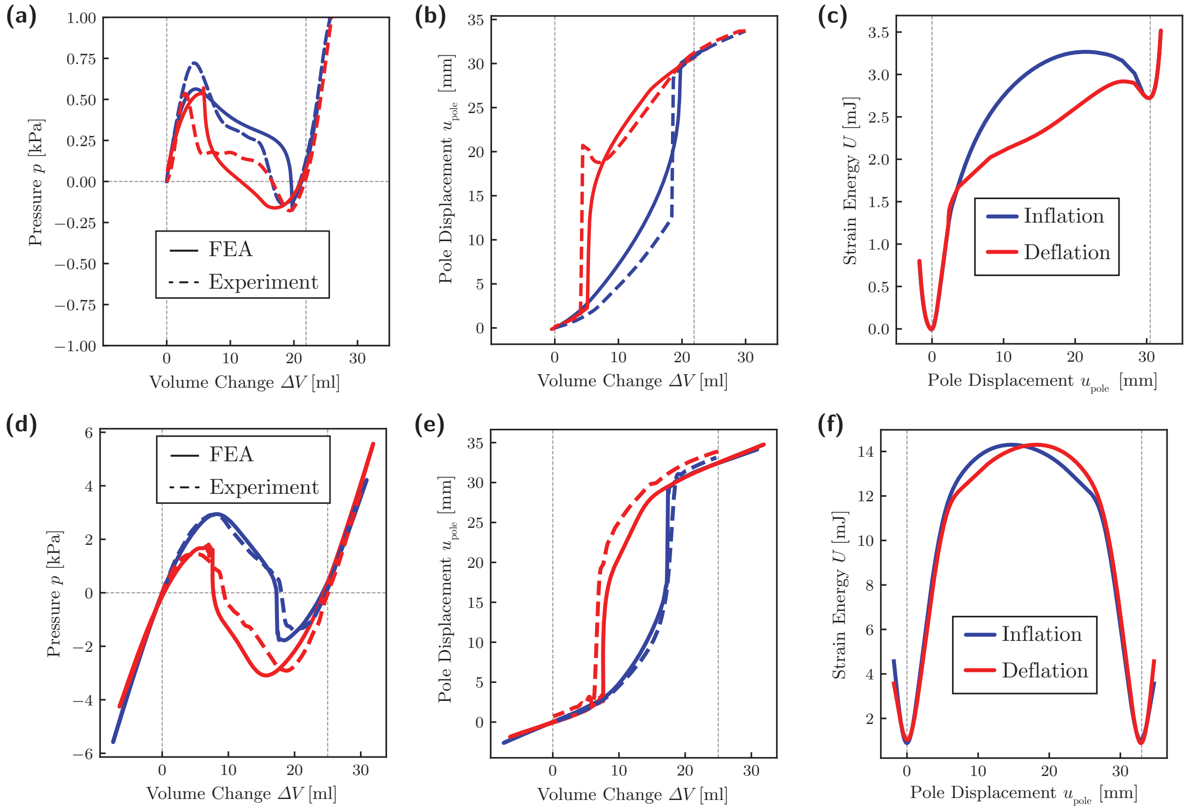

Supplementary Figure 11: Finite element simulations of the single doubly curved and universally bistable shells. The numerical results are presented as solid lines whereas the corresponding experiments are drawn as dashed lines. Blue colored lines correspond to the inflation whereas red lines correspond to the deflation of each shell. (a) Comparison between experimental and numerical pressure-volume curves for the single shell. (b) Comparison between experimental and numerical pole displacement-volume curves for the single shell. (c) Numerical strain energy profile as a function of the pole displacement for the single shell. (d) Comparison between experimental and numerical pressure-volume curves for the universally bistable shell. (e) Comparison between experimental and numerical pole displacement-volume curves for the universally bistable shell. (f) Numerical strain energy profile as a function of the pole displacement for the universally bistable shell.

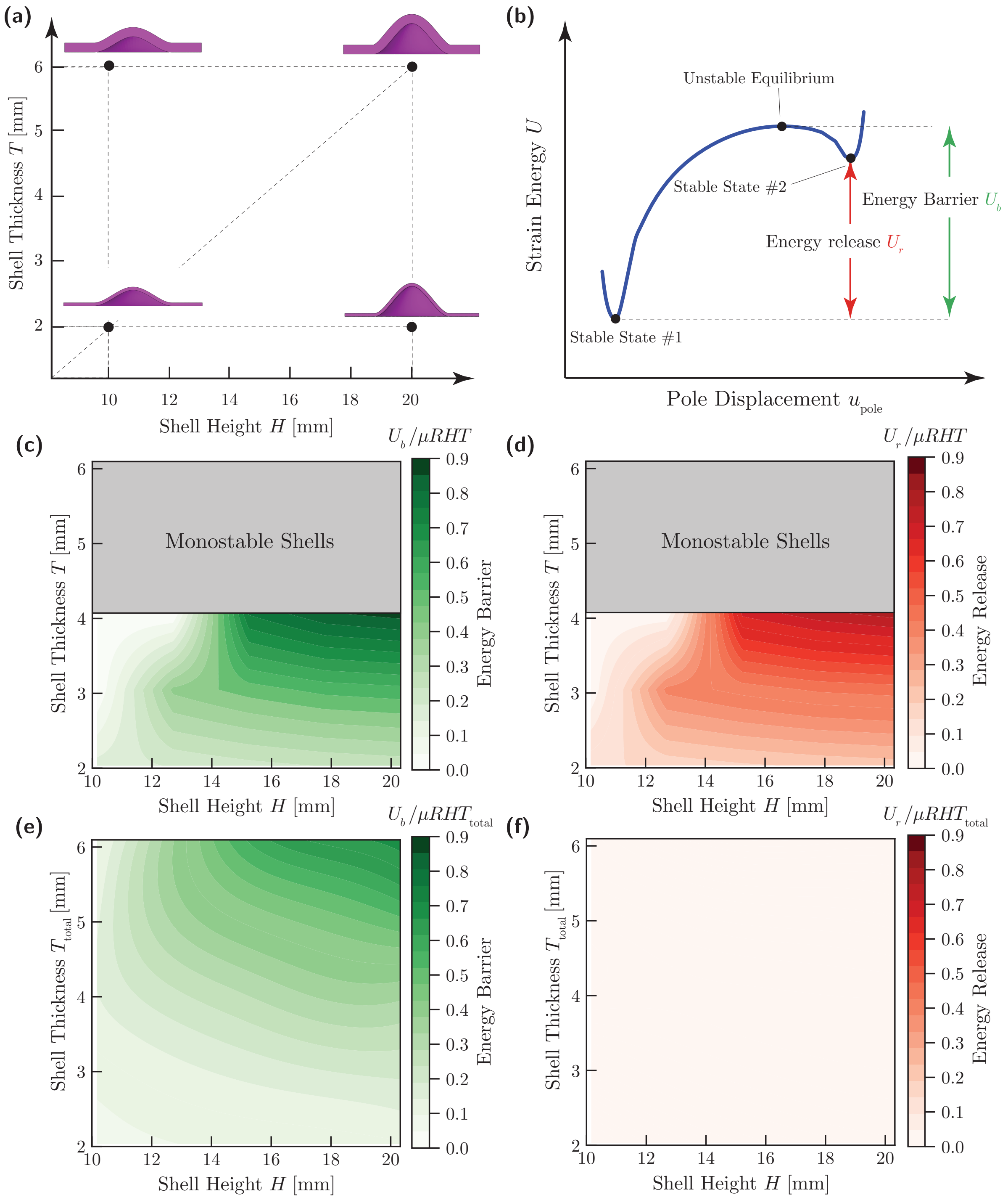

Supplementary Figure 12: Parametric Study on the effect of the height H and thickness T of single and universally bistable shells to their stability and strain energy landscape.

1.5 Shells with Tunable Energy Profile

In this section, we describe the geometry, fabrication and testing of shells with tunable energy profile.

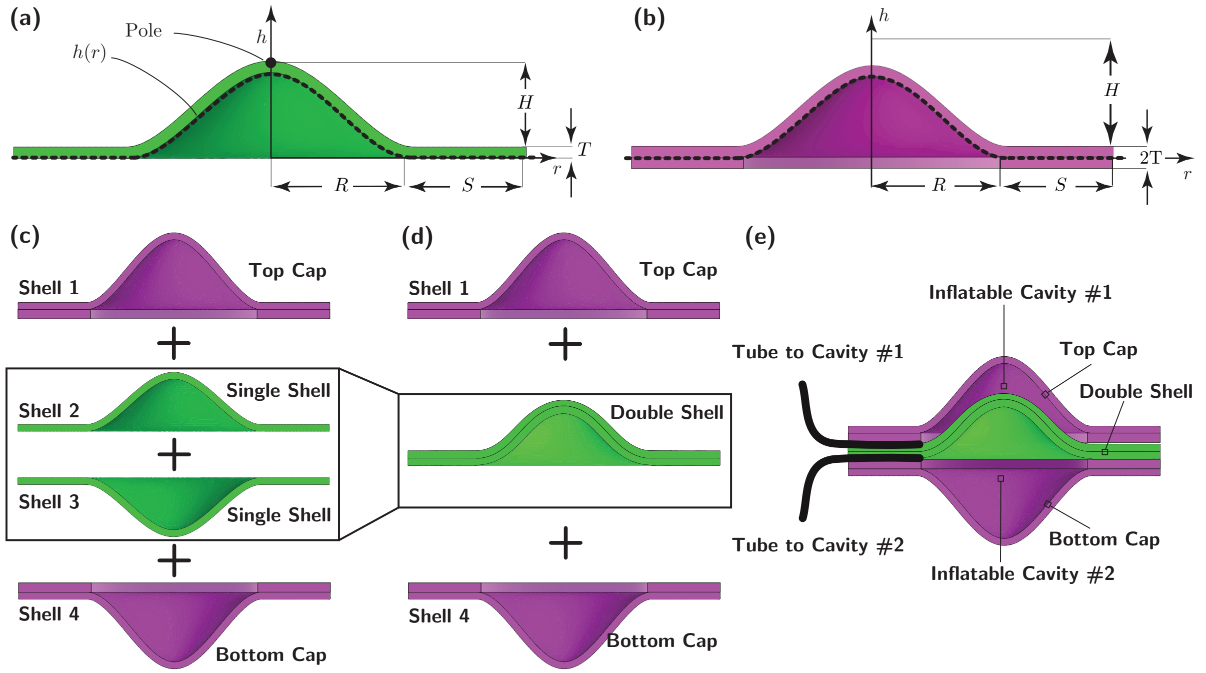

Supplementary Figure 13: Tunable shell Geometry. (a) Geometry of the single shell. (b) Geometry of the cap shell. (c) Assembly of the tunable shell from two single shells and two cap shells. (d) The two single shells are first joined to produce a double shell. (e) The final geometry of the tunable shell

1.5.1 Geometry

Our tunable shells comprise two pairs of single shells (see Fig. 13). and are obtained by first joining a pair of single shells to form a universally bistable shell (see Fig. 13c-d) witch is then sandwiched between the two remaining shells (see Fig. 13d-e). Notably, this fabrication process results in the formation of two inflatable cavities (see Fig. 13e) and the energy profile of the system can be tuned by controlling their volume.

1.5.2 Fabrication

The tunable shells fabricated and tested in this study are made of silicone rubber. Specifically, the outer single shells are made of Elite Double 8 (Zhermack), whereas the universally bistable shell sandwiched between them is made of Elite Double 32 (Zhermack).

The outer single shells were casted using the two-part mold shown in Fig. 14 which was 3d printed in Vero-clear using an Objet Connex 500 printer (Stratasys). The values of the geometric parameters used in this study for the outer shells are summarized in Table 2, whereas the geometric parameter values for the universally bistable shell (shown in green in Fig. 13b-c) are identical to the ones presented in Section 5. Note that the outer shells feature an additional rim of width S and thickness T in order to further separate them from the underlying universally bistable shells and enable the formation of the inflatable cavities.

Radius (R)	25.4mm
Height (H)	12mm
Thickness (T)	1mm
Flat Length (S)	20mm

Table 2: Values of the geometric parameters for the single shells comprising the top and bottom caps of the tunable shell.

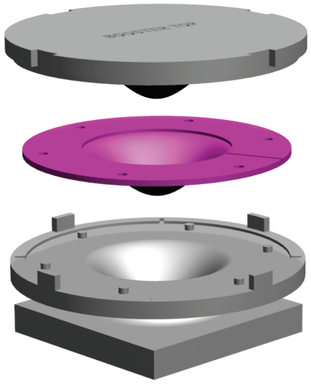

Supplementary Figure 14: The 3D printed mold used for the fabrication of the top and bottom cap shells required for the tunable shell.

The fabrication of our tunable shells consists of the following 20 steps:

Step 1: expose all inner surfaces of the mold shown in Fig. 3 to Ease Release 200 spray (Mann Release Technologies) to facilitate the process of removing the cured silicone rubber.
Step 2: pour a mixture of equal amounts of Elite Double 32 base and catalyst (here made using the Zhermack Doublemix mixer) into Part A of the mold (see Fig. 3).
Step 3: slowly place part B of the mold on top of part A, allowing for any excess silicone

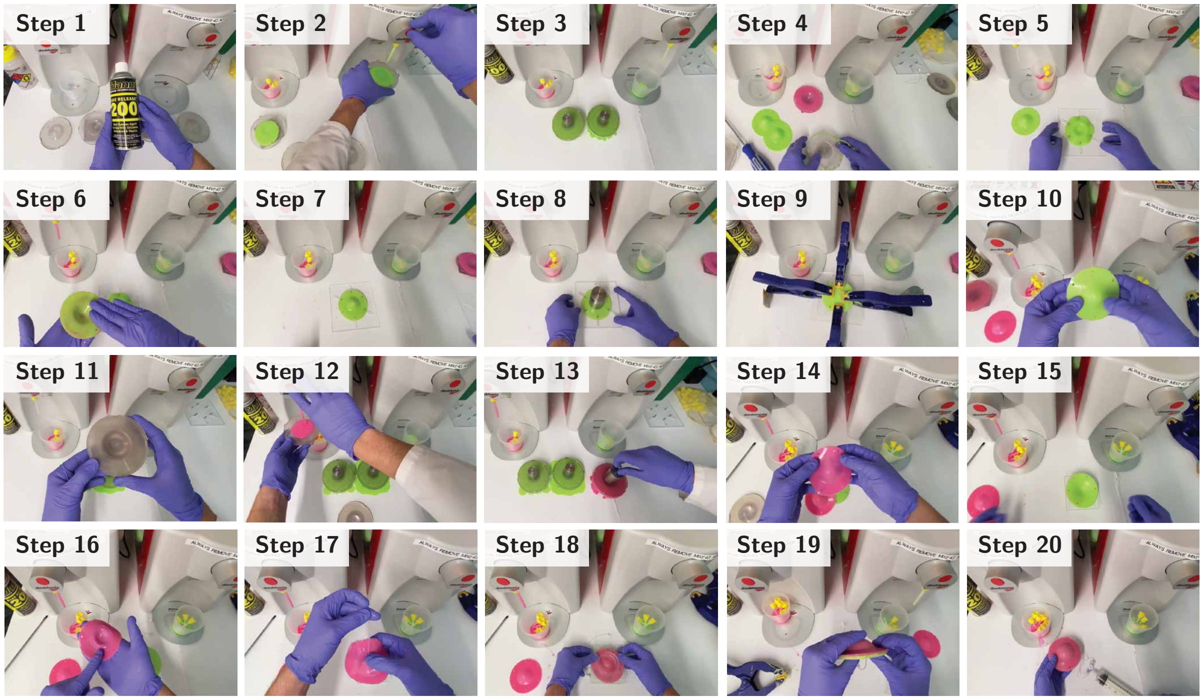

Supplementary Figure 15: The 20 Steps for the Fabrication of the tunable shells.

rubber to flow out of the mold and cure for 20 minutes.
Step 4: remove the single shell from its molds peeling off any excess silicone. Repeat the process to fabricate two shells.
Step 5: place one of the shells upside down on a flat rigid surface.
Step 6: gently spread a thin layer of Elite Double 8 on the inner surface of the second shell to glue the two shells together.
Step 7: place and align the second shell directly on top of the upside-down shell.
Step 8: flatten the two shells with a rigid plate. Place a small weight on top of the rigid surface to ensure that the two shells remain in contact while the silicone rubber cures.
Step 9: cure for 20 minutes with the two rigid plates clamped to ensure a uniform seal between the two single shells.
Step 10: remove the double shell from the rigid plates and set it aside.
Step 11: expose all inner surfaces of the mold shown in Fig. 14to Ease Release 200 spray (Mann Release Technologies) to facilitate the process of removing the cured silicone rubber.
Step 12: pour a mixture of equal amounts of Elite Double 8 base and catalyst (here made using the Zhermack Doublemix mixer) into Part A of the mold (see Fig. 14).
Step 13: slowly place part B of the mold on top of part A, allowing for any excess silicone rubber to flow out of the mold and cure for 20 minutes.
Step 14: remove the single shell from its molds peeling off any excess silicone. Repeat

the process to fabricate two shells.
Step 15: place the double shell made of Elite Double 32 upside down on a flat surface.
Step 16: gently spread a thin layer of Elite Double 8 on the outer rim of the inner surface of one of the single shells made of Elite Double 8. Avoid spreading Elite Double 8 on the region of the cavity at this point.
Step 17: align a narrow tube that will be used to inflate the cavity.
Step 18: place the single shell with the narrow tube on top of the double shell and press firmly on the outer rim region where the two shells must be glued.
Step 19: ensure that the double shell is glued to the single shell cap and that the cavity can be inflated without any leaks. Repeat the same process on the reverse side of the double shell by gluing the second single shell cap.
Step 20: ensure that both cavities can be inflated without any leaks.

1.5.3 Testing

In order to characterize the quasi-static response of our tunable shell we conducted experiments to determine its pressure-volume relationship upon inflation and deflation for different levels of pre-inflation of the two internal cavities. Specifically, we considered,
Test 1: Both Cavity 1 and Cavity 2 are not pre-inflated ($\Delta V_{p,1} = 0$ and $\Delta V_{p,2} = 0$) (see Fig. 16a)
Test 2: Cavity 1 is not pre-inflated ($\Delta V_{p,1} = 0$) and Cavity 2 is pre-inflated by supplying $\Delta V_{p,2} = 10$ml (see Fig. 16a)
Test 3: Cavity 1 is not pre-inflated ($\Delta V_{p,1} = 0$) and Cavity 2 is inflated by supplying $\Delta V_{p,2} = 20$ml (see Fig. 16a)

Note that all tests are conducted as detailed in Section 1.3 and that the results from Tests 2-3 can be used to deduce the response of the shell if Cavity 1 was pre-inflated instead of Cavity 2. This is because an inflation test with $\Delta V_{p,1} = 0$ and $\Delta V_{p,2} = 10/20$ ml is identical to a deflation test with $\Delta V_{p,1} = -10/-20$ ml and $\Delta V_{p,2} = 0$.
In Fig. 16b we report the pressure-volume curves recorded during inflation (blue lines) and deflation (red lines) for Tests 1-3. We find that for Test 1 the response of the tunable shell is qualitatively identical to the one of the universally bistable shell (see Section 1.3). However, the tunable shell requires a larger amount of pressure to transition to its second stable state (inverted) owing to the significantly stiffer rubbers used (Elite Double 32) and its overall increased thickness.

Differently, in Test 2 and Test 3 the maximum pressure required to invert the shell during inflation drops but the magnitude of the negative pressure required to bring the tunable shell back to its original state increases. This suggests that by pre-inflating Cavity 2 we increase the elastic strain energy stored in the initial state while simultaneously decreasing the elastic strain energy stored in the inverted state of the tunable shell. As a result, in Test 3 only a small input pressure is required to invert the tunable shell and

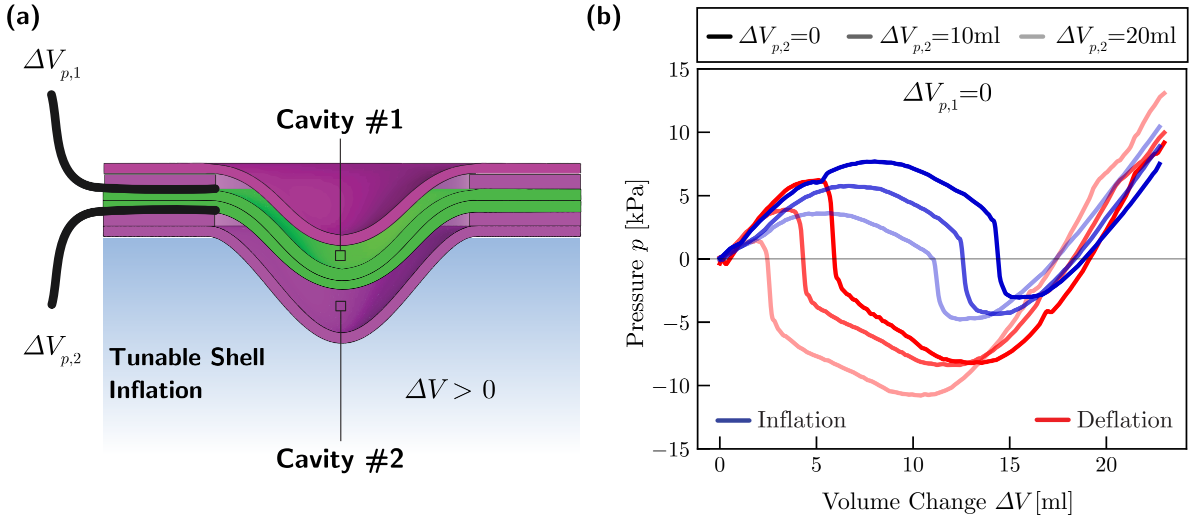

Supplementary Figure 16: Testing of the tunable shell. (a) Schematic of the tunable shell. (b) The experimental pressure-volume relationship for the tunable shell for 3 different levels of pre-inflation for Cavity 2.

release a large amount of stored elastic energy.

Supplementary Note 2: Arrays of Universally Bistable Shells

In this section we discuss the experimental setup and provide details for the testing and modeling of transition waves that propagate in arrays of universally bistable shells.

2.1 Fabrication

The experimental setup used to propagate transition waves in arrays of universally bistable shells is shown in Fig. 17. This setup consists of a 1D array of universally bistable shells, acrylic tubes and plates to connect them, two pressure regulators, a solenoid valve, an LCD screen, an Arduino and a power supply.

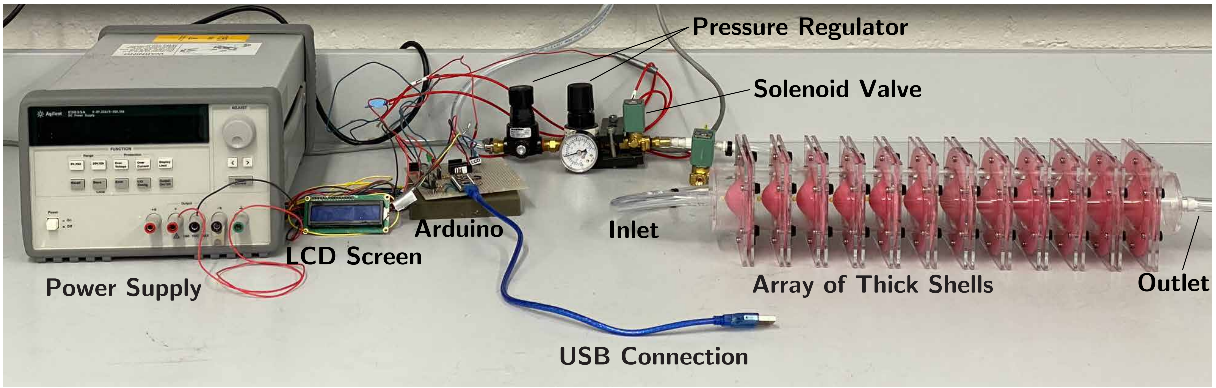

Supplementary Figure 17: The experimental setup used to study the propagation of transition waves in arrays of universally bistable shells.

To construct the array of universally bistable shells we glue clear cast acrylic tubes (McMaster Carr part number: 8532K46) of length L_t and internal radius[1] $R = 25.4$mm to acrylic plates (McMaster Carr part number: 1178T12) and sandwich the universally bistable shells between them as shown in Fig. 18. To hold the universally bistable shell in place and eliminate any leaks we connect the two acrylic plates adjacent to each universally bistable shell using stainless steel M3 screws (McMaster Carr part number: 90751A113) and thumb nuts (McMaster Carr part number: 93886A220). Tesselating this combination of acrylic plates, tubes and universally bistable shells leads to the array of universally bistable shells depicted in Fig. 17.

When the array is assembled, the acrylic tubes used to connect the universally bistable shells, enclose a finite volume of air equal to $V_{\text{air}} = \pi R^2 L_t$ at atmospheric pressure, where R is the internal radius of the acrylic tubes and L_t is their length (see Fig. 18). Any relative displacement between the universally bistable shells adjacent to each acrylic tube

[1]Note that the internal radius of the tubes is identical to the radius of the universally bistable shells

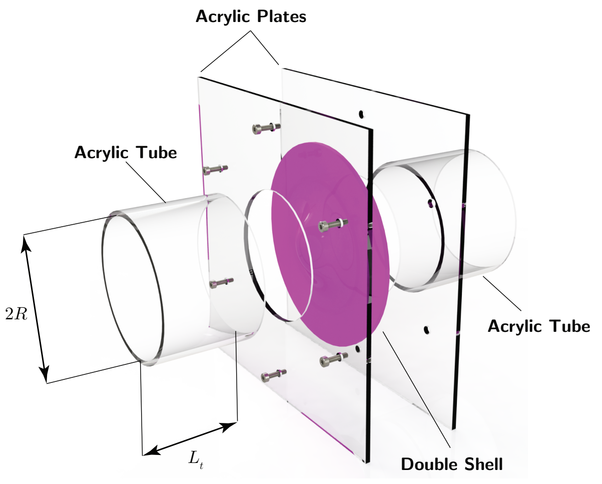

Supplementary Figure 18: Schematic highlighting the details of the connections between the acrylic tube, acrylic plates and the universally bistable shells in order to construct the arrays of universally bistable shells.

changes the volume of the air enclosed by the tube resulting in a pressure/force which in turn acts on the shells resisting the enforced volume change. As a result, the array of universally bistable shells connected via acrylic tubes, forms a closed system of bistable springs (i.e. universally bistable shells) coupled via interconnecting springs (i.e. air cavity of each acryclic tubes). Having established the bistability of the universally bistable shells used in the array, we study the transition waves that propagate in such arrays when one of the shells is pushed from one stable state to another.

2.2 Testing

In order to provide energy to the array of universally bistable shells and propagate transition waves, we connect the first shell in the array to a pressure supply. To precisely control the excitation pressure provided to the array, we decreased the pressure from the wall air-outlet (∼200psi) using two pressure regulators (1/4 NPT 15CFM by Wilkerson and 1/4 NPT 9CFM by Coilhose Pneumatics) connected in series. The first pressure regulator reduces the inlet pressure from ∼200psi to ∼ 40psi, whereas the second one accurately controls the pressure in the range [0,40]psi. Further, to turn on and off the input pressure we used a standard two-way solenoid valve (SC8256A002V - ASCO). The solenoid valve was powered through an external power supply using 9V and 0.1A and controlled via an NPN transistor (IRF520 - Vishay Siliconix). In all of our tests, we provided pressure to the first shell of the array for 100ms after which point the input pressure was reduced down to atmospheric.

To capture the deformation of each shell and the propagation of the transition waves in our system, we used a digital camera (Sony RX100 IV) capable of slow motion video

at 480 frames per second (×20 slower). Specifically, we glued two colored pins (a yellow one and a green one - see Fig. 19a-b) to each shell and determined the displacement of the poles by tracking them. The yellow pins were visible when the shells were at their first stable state (see Fig. 19a) hiding the corresponding green pins. Differently, when the shells were inverted to their second stable state, only the green pins were visible (see Fig. 19b) hiding the corresponding yellow pins.

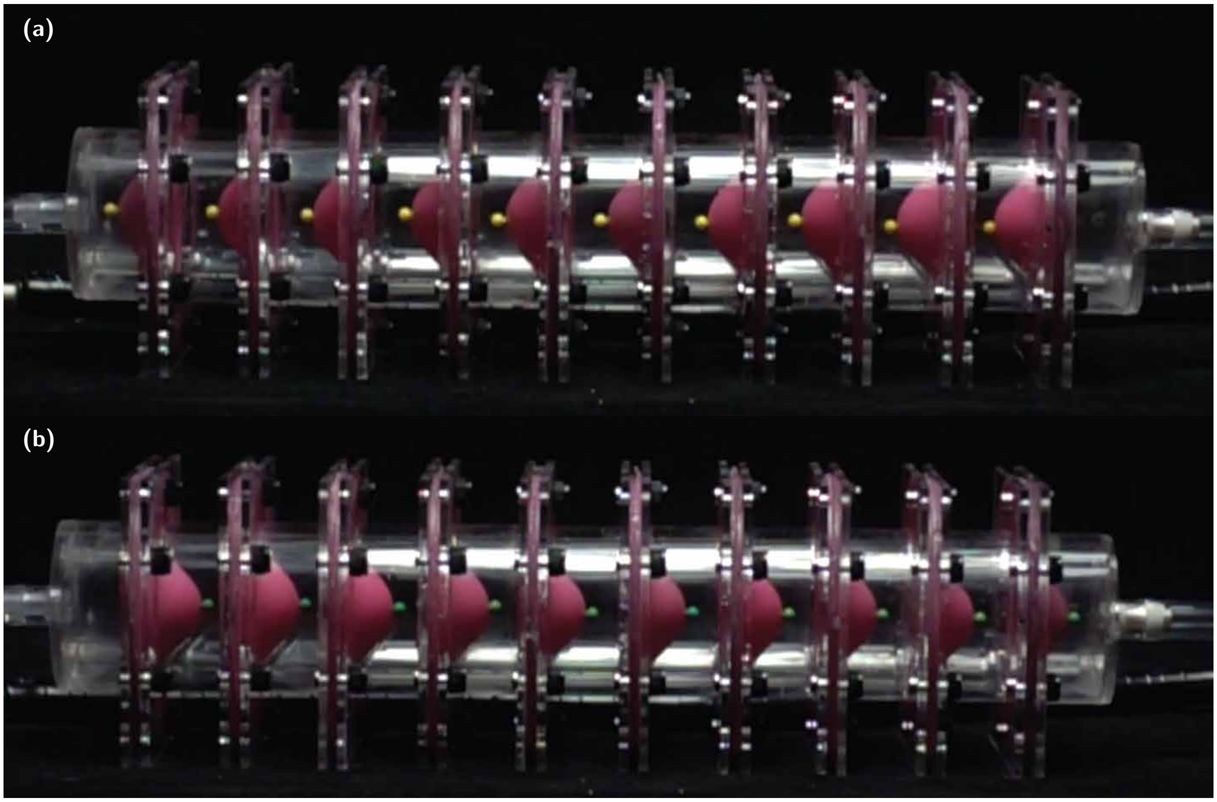

Supplementary Figure 19: Snapshots of the array of universally bistable shells highlighting the two different colored pins used to track the displacements of each shell. (a) All shells are at their first stable state. (b) All shells are at their second stable state

2.3 Discrete Model

To better understand the propagation of transition waves in our arrays of universally bistable shells connected via acrylic tubes we develop a discrete model. To this end, we focus on the $[i]$-th shell in the system, with mass m, strain energy potential U_i connected to shells $[i-1]$ and $[i+1]$ through acrylic tubes with length L_t (see Fig. 20a). To determine the equation of motion for the $[i]$-th shell in the system we assume that,

(i) the mass m is concentrated at the pole of the shell.

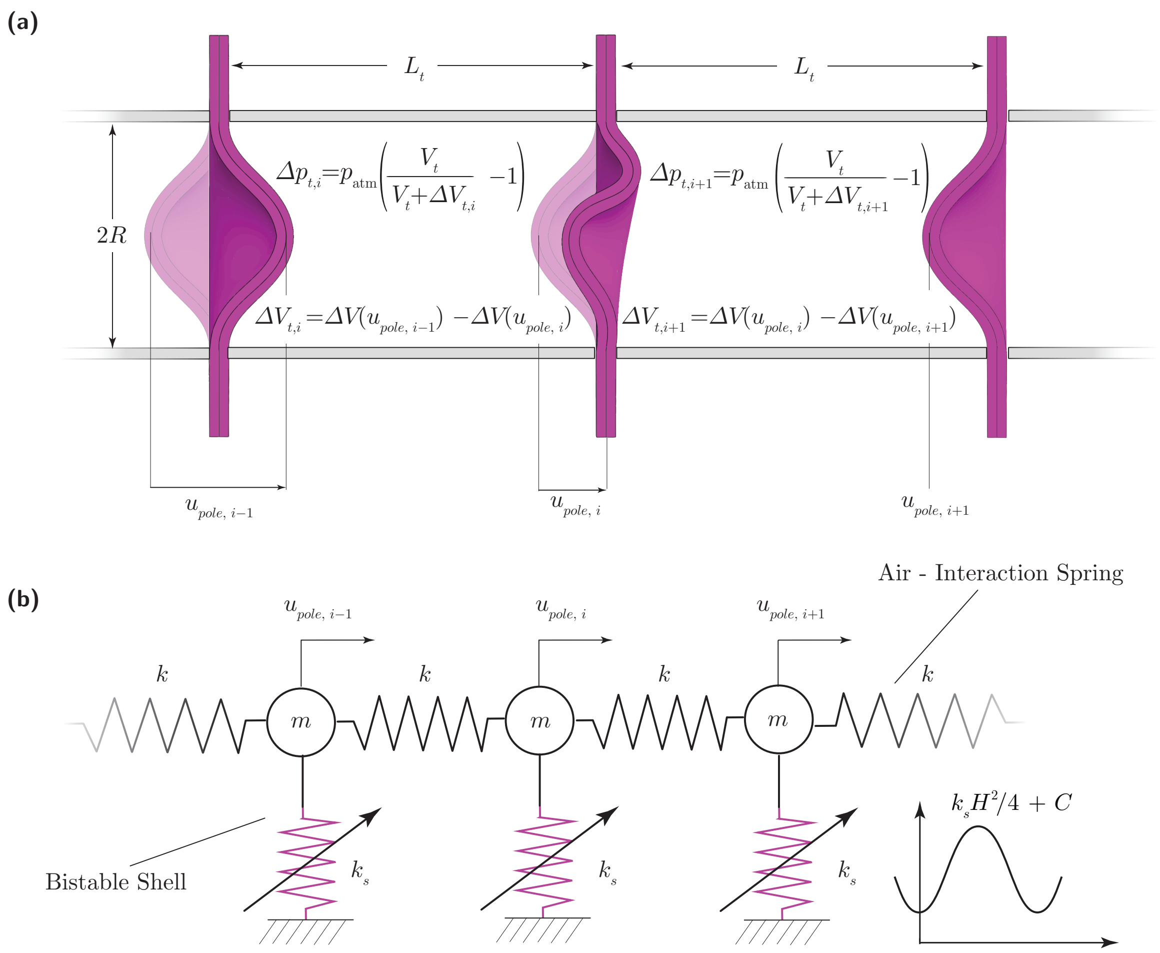

Supplementary Figure 20: Array of universally bistable shells. (a) Schematic of the pole displacement and corresponding volume change for the $[i-1]$, $[i]$ and $[i+1]$-th shell in the array. (b) Simplified approximation of the system as a discrete collection of masses, bistable springs and interconnecting springs.

(ii) the kinematics of the shell are fully captured by its pole displacement $u_{\text{pole},i}$.

(iii) a displacement of $u_{\text{pole},i}$ leads to a corresponding volume change of the air adjacent to the $[i]$-th shell equal to $\Delta V_i(u_{\text{pole},i})$. Note, that the relationship between the change in volume of each shell ΔV_i and its pole displacement $u_{\text{pole},i}$ can be determined from our quasi-static experiments and FEA simulations (see Fig. 8 and Fig. 11).

(iv) the change in the volume of air $\Delta V_{t,i}$ enclosed by tube i, connecting shells $[i-1]$ and $[i]$ (see Fig. 20a) can be computed from their corresponding changes in volume

ΔV_{i-1} and ΔV_i through,

$$\Delta V_{t,i} = \Delta V_i \left(u_{\text{pole},i}\right) - \Delta V_{i-1} \left(u_{\text{pole},i-1}\right) \tag{4}$$

(v) changes in the volume of air enclosed by tube $[i]$, $\Delta V_{t,i}$, results in a a pressure change Δp_i that can be determined via Boyle's law

$$p_{\text{atm}} V_0 = \left(p_{\text{atm}} + \Delta p_i\right) \left(V_0 + \Delta V_{t,i}\right) \tag{5}$$

where $V_0 = \pi R^2 L_t$ is the initial volume of air enclosed by tube $[i]$ and p_{atm} is the initial (atmospheric) pressure. It follows from Eq. (5) that

$$\Delta p_i = p_{\text{atm}} \left(\frac{\pi R^2 L_t}{\pi R^2 L_t + \Delta V_{t,i}} - 1 \right) \tag{6}$$

Under these assumptions, the equation of motion for the $[i]$-th shell can be written as

$$m \frac{d^2 u_{\text{pole},i}}{dt^2} + \beta \frac{d u_{\text{pole},i}}{dt} + \frac{dU\left(u_{\text{pole},i}\right)}{d u_{\text{pole},i}} + f_{i-1} - f_i = 0 \tag{7}$$

where U is the quasi-static bistable strain energy potential for the universally bistable shells, β is a linear damping parameter and f_{i-1} and f_i are the forces acting on the $[i]$-th shell due to the changes in volume in tube $[i-1]$ and $[i]$, respectively. Such forces can be determined from Eq. (6) as

$$\begin{aligned} f_i &= \pi R^2 p_{\text{atm}} \left(\frac{\pi R^2 L_t}{\pi R^2 L_t + \Delta V_{i+1}(u_{\text{pole},i+1}) - \Delta V_i(u_{\text{pole},i})} - 1 \right), \\ f_{i-1} &= \pi R^2 p_{\text{atm}} \left(\frac{\pi R^2 L_t}{\pi R^2 L_t + \Delta V_i(u_{\text{pole},i}) - \Delta V_{i-1}(u_{\text{pole},i-1})} - 1 \right) \end{aligned} \tag{8}$$

Substitution of Eqs. (8) into Eq. (7) yields

$$\begin{aligned} & m \frac{d^2 u_{\text{pole},i}}{dt^2} + \beta \frac{d u_{\text{pole},i}}{dt} + \frac{dU\left(u_{\text{pole},i}\right)}{d u_{\text{pole},i}} + \\ & \pi R^2 p_{\text{atm}} \left[\frac{\pi R^2 L_t \left(\Delta V_{i+1} - 2\Delta V_i + \Delta V_{i-1}\right)}{\left(\Delta V_{i+1} - \Delta V_i + \pi R^2 L_t\right)\left(\Delta V_i - \Delta V_{i-1} + \pi R^2 L_t\right)} \right] = 0. \end{aligned} \tag{9}$$

For a system of N universally bistable shells, Eq. (9) results in a system of N coupled differential equations, which given the strain energy potential $U(u_{\text{pole}})$ and pole displacement-volume $\Delta V(u_{\text{pole}})$ relationships can be solved numerically to determine the pole displacement of the $[i]$-th universally bistable shell as a function of time t.

In this study, we consider arrays comprising 10 universally bistable shells and use a Python implementation of the Dormand Prince 8(5,3) method to integrate Eqs. (9). The mass of each universally bistable shell m was taken to be the average of each shell in the array and was found equal to $m = 30$g. In every time step, given $u_{\text{pole},i}$, we use our FE results shown in Fig. 11 to determine ΔV_i and $dU_i/du_{\text{pole},i}$. Moreover, we initiate transition waves by controlling the pole displacement of the first shell through,

$$u_{\text{pole},1} = H\left[1 \pm \tanh\left(f(t - t_0)\right)\right] \tag{10}$$

where H is the height of the universally bistable shell[2], f is the characteristic frequency of the pulse (in shells/s), t is time and t_0 is the time at which the pulse arrives at the first shell. Note that in all our numerical analyses we use $\beta = 2.5$kg/s - value obtained by fitting the result of our discrete model to a single experiment on the same system.

2.3.1 Results

To verify the validity of our discrete model, in Fig. 21 we compare experimental (markers) and numerical (solid lines) results for three different tests. Specifically, in Figs. 21a-b we consider an array of 10 universally bistable shells in which the wave is initiated both at the first (Figs. 21a) and last (Figs. 21b) unit, resultig in a left-to-right and right-to-left propagation, respectively. Further, in Fig. 21c we present results for an array of 12 universally bistable shells. In all three experimental tests, transition waves are initiated by providing a rectangular pressure pulse supplying $\Delta p = 69kPa$ for 100ms. In all three simulations, the arrays are excited using a pulse with the form of Eq. (10) with frequency $f = 43.4$Hz and start time $t_0 = 0.136$s. The transition waves of Fig. 21a and Fig. 21c are "left-to-right" and are initiated by prescribing the displacement of the first shell in the array through,

$$u_{\text{pole},1} = H\left[1 + \tanh\left(43.4(t - 0.136)\right)\right] \tag{11}$$

In contrast, the transition wave of Fig. 21b is "right-to-left" and is initiated by prescribing the displacement of the last shell in the array through,

$$u_{\text{pole},10} = H\left[1 - \tanh\left(43.4(t - 0.136)\right)\right] \tag{12}$$

All plots clearly indicate that the discrete model is capable of capturing the response of observed in experiments, as suggested by the great agreement between the two. Note that due to the inversion of each universally bistable shell during the propagation of transition waves, the bright colored markers used for image processing our experimental results are naturally not visible for a range of pole displacement values resulting to the lack of experimental data in the pole displacement range $[10, 20]$mm. Further, the results

[2]Note that the height of the universally bistable shell coincides with half of the pole displacement between the two stable states.

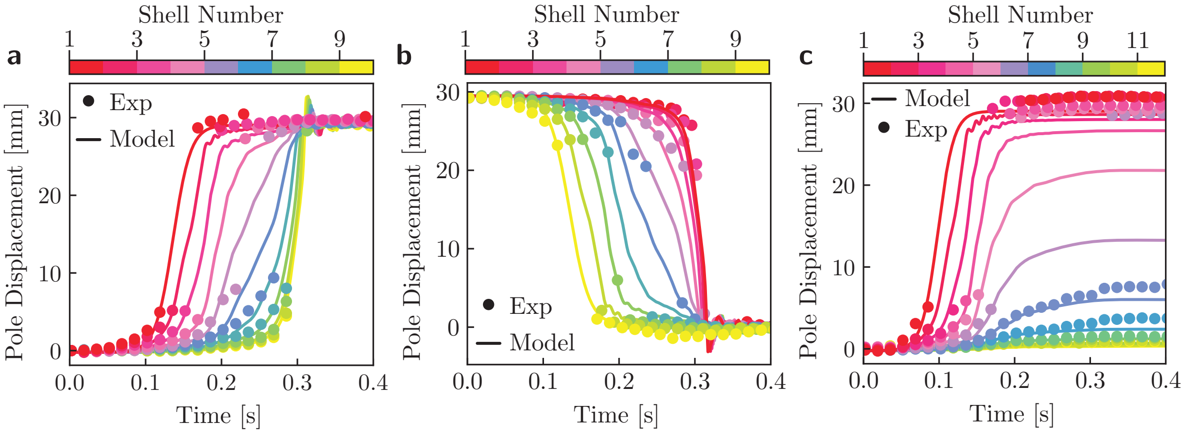

Supplementary Figure 21: Results of the discrete model and comparison against experiments for a pulse with frequency $f = 43.4$Hz and start time $t_0 = 0.136$s . Solid lines represent the discrete model predictions whereas markers correspond to experimental measurements. (a) Array of 10 universally bistable shells in which the wave initiates from the first shell in the array. (b) Array of 10 universally bistable shells in which the wave initiates from the last shell in the array. (c) Array of 12 universally bistable shells in which the wave initiates from the first shell in the array

of Fig. 21a-b indicate the capability of our system to support bidirectional transition waves, since in both cases, using the same input energy, the transition wave arrives at the end of the array with exactly the same characteristics, while being initiated from opposite sides of the same array. The results of Fig. 21c suggest that transition waves cannot fully propagate in arrays of 12 universally bistable shells, since due to the effects of dissipation, only six shells transition from their initial to their inverted stable state. In this case, despite performing considerably worse than in cases where the waves fully propagate through the array, our discrete model can still capture the main features of the partial transition wave.

Finally, in Fig. 22 we compare the discrete model predictions to our experimental measurements for forward (left to right) and backward (right to left) pulses initiated using 10, 15, 20 and 25 psi of pressure, respectively. The results further demonstrate our system's ability to support bidirectional transition waves, but also indicate that the input energy can affect the effective transition wave speed.

2.4 Continuum Model

To gain a deeper insight into the propagation of transition waves in our system, we next seek analytical solutions to Eqs. (9) by taking their continuum limit in the absence of dissipative phenomena. We begin by approximating the interaction forces between

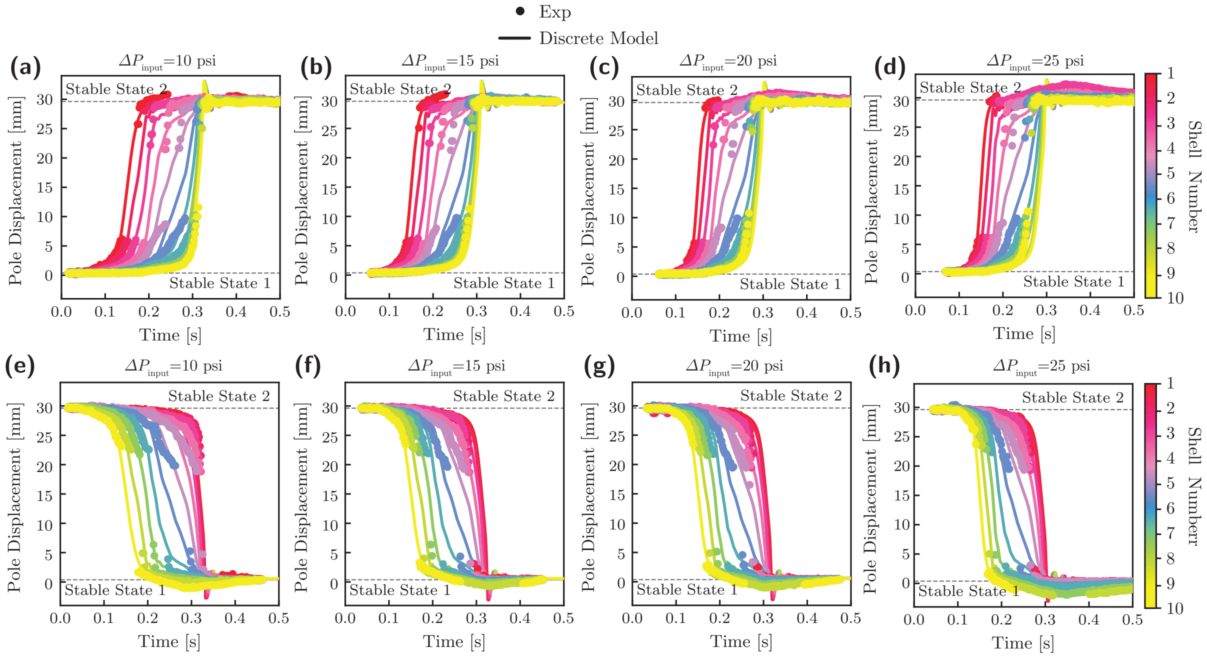

Supplementary Figure 22: Results of the discrete model and comparison against experiments for forwards and backwards propagation of transition waves initiated by pulses of increasing energy. Solid lines represent the discrete model predictions whereas markers correspond to experimental measurements. (a)-(d) Forward propagation for pulses initiated using (a) 10, (b) 15, (c) 20 and (d) 25 psi of pressure. (e)-(h) Backward propagation for pulses initiated using (e) 10, (f) 15, (g) 20 and (h) 25 psi of pressure.

adjacent shells, f_i and f_{i-1} (given by Eq. (8)) as

$$f_i \approx k_i \left(u_{\text{pole},i} - u_{\text{pole},i+1}\right), \qquad f_{i-1} \approx k_{i-1} \left(u_{\text{pole},i-1} - u_{\text{pole},i}\right) \tag{13}$$

where the linear interaction stiffnesses k_i, k_{i-1} are obtained by performing a least squares fit to the fully nonlinear interaction force-relative pole displacement curve as shown in Fig. 23.

Using Eq. (13) to represent the interaction forces between shells and in absence of viscous dissipation effects (i.e. $\beta = 0$), the governing equation of motion for the $[i]$-th shell takes the form,

$$m\frac{d^2 u_{\text{pole},i}}{dt^2} + \frac{dU(u_{\text{pole},i})}{du_{\text{pole},i}} - k\left(u_{\text{pole},i+1} - 2u_{\text{pole},i} + u_{\text{pole},i-1}\right) = 0. \tag{14}$$

Next, we take the continuum limit of Eq.14. To this end, we introduce the continuous function $u\left(\tilde{x},\, t\right)$ that interpolates the pole displacement $u_{\text{pole},i}$ as

$$u\left(\tilde{x} = i, t\right) = u_{\text{pole},i}, \tag{15}$$

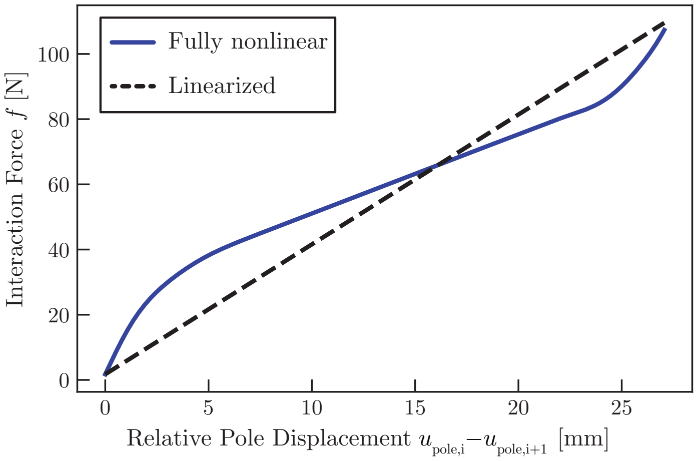

Supplementary Figure 23: Comparison between the fully nonlinear interaction forces used in our discrete model (Eq. (8)) and the linearized approximation used in our discrete model

where $\tilde{x} = x/L_t$ is the normalized coordinate along the x-axis. If the width of the propagating waves is much larger than the unit shell-to-shell distance, the pole displacements of the $[i-1]$-th and $[i+1]$-th shell can then be expressed via Taylor expansion as,

$$\begin{aligned} u_{\text{pole},i-1} &= u\left(i-1,\, t\right) = \left[u - \frac{\partial u}{\partial \tilde{x}} + \frac{1}{2}\frac{\partial^2 u}{\partial \tilde{x}^2}\right]_{\tilde{x}=i} \\ u_{\text{pole},i+1} &= u\left(i+1,\, t\right) = \left[u + \frac{\partial u}{\partial \tilde{x}} + \frac{1}{2}\frac{\partial^2 u}{\partial \tilde{x}^2}\right]_{\tilde{x}=i} \end{aligned} \tag{16}$$

Substituting Eqs.(15) and (16) into Eq. (14) yields the continuum governing equation,

$$\frac{\partial^2 u}{\partial t^2} + \frac{1}{m}\frac{dU(u)}{du} - c_0^2 \frac{\partial^2 u}{\partial x^2} = 0 \tag{17}$$

where $c_0 = \sqrt{k/m} = 364.44$ shells/s is the ratio of the interaction spring stiffness to the mass of each shell. Finally, we introduce the travelling coordinate $\zeta = \tilde{x} - ct$ and apply the chain rule to recompute the temporal and spatial derivatives as,

$$\frac{\partial^2(\cdot)}{\partial t^2} = c^2 \frac{\partial^2(\cdot)}{\partial \zeta^2}, \qquad \frac{\partial^2(\cdot)}{\partial \tilde{x}^2} = \frac{\partial^2(\cdot)}{\partial \zeta^2} \tag{18}$$

Substitution of Eq. (17) into Eqs. (18) yields

$$(c_0^2 - c^2)\frac{\partial^2 u}{\partial \zeta^2} = \frac{1}{m}\frac{dU(u)}{du}. \tag{19}$$

In an effort to obtain analytical solutions to Eq. (19) we assume that the bistable energy potential for the universally bistable shells $U(u)$ can be approximated with the following 4th order polynomial

$$U(u) \approx \frac{1}{4} k_s u^2 \left(\frac{u}{H} - 2 \right)^2 + C \tag{20}$$

where H is the height of the universally bistable shell. As shown in Fig. 24, this polynomial describes an energy profile with two identical stable states at $u = 0$ (initial state of the shell) and $u = 2H$ (inverted state of the shell)

$$U(0) = U(2H) = C \tag{21}$$

separated by an energy barrier of magnitude $k_s H^2/4$.

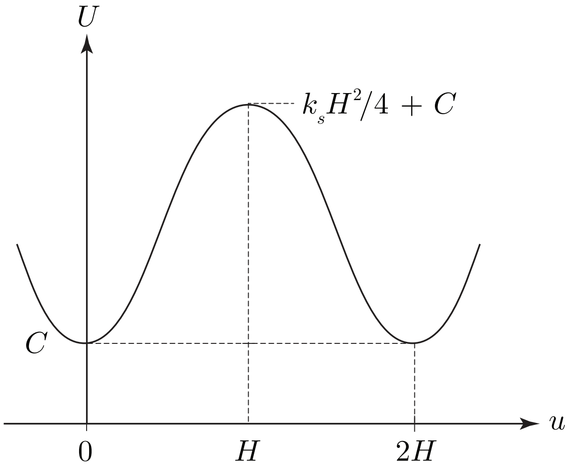

Supplementary Figure 24: The 4th order polynomial approximation to the strain energy potential of the universally bistable shells.

By substituting Eq. (20) into Eq. (19) we obtain

$$\frac{\partial^2 u}{\partial \zeta^2} = \frac{c_s^2}{c_0^2 - c^2} u \left(\frac{u}{H} - 1 \right) \left(\frac{u}{H} - 2 \right) \tag{22}$$

where $c_s = \sqrt{k_s/m} = 90.87$ shells/s. Remarkably, Eq. (22) has the form of Klein-Gordon Equation with quadratic and cubic non-linearties and has been shown to admit an analytical solution with form [?]

$$u = H \left[1 \pm \tanh \left(\frac{\zeta}{w} \right) \right] = H \left[1 \pm \tanh \left(\frac{x - ct}{w} \right) \right] \tag{23}$$

where c and w are the speed and width of the propagating transition waves. Next, we determine c and w as a function of the geometry of the system and the energy supplied

to the first unit to initiate the pulse. First, we substitute the solution (23) into Eq. (22) and find that the latter is identically satisfied only if

$$w = \sqrt{\frac{2(c_0^2 - c^2)}{c_s^2}}. \tag{24}$$

Second, we calculate the total energy (i.e. the summation of kinetic, interaction, bistable potential) carried by the transition waves defined by Eq. (23)

$$E = \int_{-\infty}^{\infty} \left[\frac{1}{2} m \left(\frac{\partial u(x,t)}{\partial t} \right)^2 + \frac{1}{2} k \left(\frac{\partial^2 u(x,t)}{\partial \tilde{x}^2} \right) + U(u(x,t)) \right] dx =$$

$$= H^2 \left[\frac{2}{3w}(k + mc^2) + \frac{1}{3} w k_s \right] \tag{25}$$

Since in the absence of dissipation E is equal to the energy supplied to the first unit to initiate the pulse, E_{in}, we find that

$$E_{\text{in}} = H^2 \left[\frac{2}{3w}(k + mc^2) + \frac{1}{3} w k_s \right] \tag{26}$$

which can be solved numerically to obtain c as a function of E_{in}. Further, to obtain an explicit expression for c as a function of E_{in}, we take a Taylor's series expansion of Eq. (26) around $c/c_0 = 0$ (since in our system $c/c_0 \sim 0.2$), while retaining terms up to the third order. This yields

$$E_{\text{in}} = \frac{\sqrt{2} k H^2 c_s}{3 c_0} \left(2 + \frac{c^2}{c_0^2} \right), \tag{27}$$

from which we get

$$c = \sqrt{2} c_0 \sqrt{\frac{E_{\text{in}}}{E_{\text{min}}} - 1}, \tag{28}$$

where

$$E_{\text{min}} = \frac{2\sqrt{2}}{3} H^2 \sqrt{k_s k}, \tag{29}$$

represents the minimum amount of input energy required to initiate the transition wave. Eq. (28) reveals a remarkable property of our system, namely the ability to tune the speed of the propagating transition waves by modifying the amount of energy supplied to the system. Further, it shows that the propagating velocity c increases rapidly with the input energy.

2.4.1 Predictions for dissipative systems

Despite the fact that our continuum model was developed assuming no dissipation, it can also be used to predict the finite propagation distance in systems with a nonzero dissipation (i.e. with $\beta > 0$). To this end, we estimate the energy dissipated by each shell during the propagation of the pulse, E_{damped}, as

$$E_{\text{damped}} = \int_{-\infty}^{\infty} \beta \left(\frac{\partial u}{\partial t}\right)^2 dt = c \int_{-\infty}^{\infty} \beta \left(\frac{\partial u}{\partial \zeta}\right)^2 d\zeta = \frac{4\beta c H^2}{w}. \tag{30}$$

By introducing Eq. (24), Eq. (30) can be rewritten as

$$E_{\text{damped}} = \frac{2\sqrt{2}\beta H^2 c_s c}{\sqrt{c_0^2 - c^2}}, \tag{31}$$

which, by taking a Taylor's series expansion around $c/c_0 = 0$ and retaining terms up to the second order, can be further simplified to

$$E_{\text{damped}} \approx \frac{2\sqrt{2}\beta H^2 c_s c}{c_0} \tag{32}$$

Finally, introduction of Eq. (28) into Eq. (32) yields

$$E_{\text{damped}} = 4\beta H^2 c_s \sqrt{\frac{E_i}{E_{\min}} - 1}, \tag{33}$$

where E_i denotes the energy carried by the transition wave when propagating through the i-th unit (note that in Eq. (28) we have changed E_{in} into E_i).

Next, use energy conservation to relate E_i to E_{damped} as

$$E_{i+1} - E_i = -E_{\text{damped}} = -4\beta H^2 c_s \sqrt{\frac{E_i}{E_{\min}} - 1}. \tag{34}$$

To solve Eq. (34) and determine the number of units that the wave switches before stopping, N_{stop}, we define a continuous function $E(\tilde{x})$ that interpolate the discrete values of E_i as

$$E(\tilde{x} = i) = E_i \tag{35}$$

By introducing the continuum function Eq. (34) can be rewritten as,

$$\frac{dE}{d\tilde{x}} = -4\beta H^2 c_s \sqrt{\frac{E}{E_{\min}} - 1} \tag{36}$$

or, alternatively

$$\frac{dE}{\sqrt{\frac{E}{E_{\min}} - 1}} = -4\beta H^2 c_s d\tilde{x}. \tag{37}$$

Next, we integrate both sides of Eq. (37) to obtain

$$\int_{E_0}^{E_{N_{\rm stop}}} \frac{dE}{\sqrt{\frac{E}{E_{\rm min}}-1}} = -\int_0^{N_{\rm stop}} 4\beta H^2 c_s d\tilde{x} \Rightarrow 2E_{\rm min}\sqrt{\frac{E}{E_{\rm min}}-1}\Bigg|_{E_0}^{E_{N_{\rm stop}}} = -4\beta H^2 c_s N_{\rm stop}, \tag{38}$$

Since $E_0 = E_{\rm in}$ and $E_{N_{\rm stop}} = E_{\rm min}$, $N_{\rm stop}$ can be obtained from Eq. (38) as

$$N_{\rm stop} = \frac{E_{\rm min}}{2\beta H^2 c_s}\sqrt{\frac{E}{E_{\rm min}}-1}. \tag{39}$$
